# Low Power Wide Area Network, Cognitive Radio and the Internet of Things: Potentials for Integration

**DOI:** 10.3390/s20236837

**Published:** 2020-11-30

**Authors:** Adeiza J. Onumanyi, Adnan M. Abu-Mahfouz, Gerhard P. Hancke

**Affiliations:** 1 Department of Electrical, Electronic and Computer Engineering, University of Pretoria, Pretoria 0028, South Africa; adeiza1@futminna.edu.ng (A.J.O.); gp.hancke@cityu.edu.hk (G.P.H.); 2 Council for Scientific and Industrial Research (CSIR), Pretoria 0001, South Africa; 3 Department of Computer Science, City University of Hong Kong, Hong Kong, China

**Keywords:** challenges, cognitive radio, future direction, LPWAN, Internet of Things, survey

## Abstract

The Internet of Things (IoT) is an emerging paradigm that enables many beneficial and prospective application areas, such as smart metering, smart homes, smart industries, and smart city architectures, to name but a few. These application areas typically comprise end nodes and gateways that are often interconnected by low power wide area network (LPWAN) technologies, which provide low power consumption rates to elongate the battery lifetimes of end nodes, low IoT device development/purchasing costs, long transmission range, and increased scalability, albeit at low data rates. However, most LPWAN technologies are often confronted with a number of physical (PHY) layer challenges, including increased interference, spectral inefficiency, and/or low data rates for which cognitive radio (CR), being a predominantly PHY layer solution, suffices as a potential solution. Consequently, in this article, we survey the potentials of integrating CR in LPWAN for IoT-based applications. First, we present and discuss a detailed list of different state-of-the-art LPWAN technologies; we summarize the most recent LPWAN standardization bodies, alliances, and consortia while emphasizing their disposition towards the integration of CR in LPWAN. We then highlight the concept of CR in LPWAN via a PHY-layer front-end model and discuss the benefits of CR-LPWAN for IoT applications. A number of research challenges and future directions are also presented. This article aims to provide a unique and holistic overview of CR in LPWAN with the intention of emphasizing its potential benefits.

## 1. Introduction

The Internet of Things (IoT) is defined as a communication network that connects things with naming, sensing, and processing abilities [1]. Specifically, the International Telecommunication Union (ITU) refers to these things as objects of the physical world (that is, physical things) or the information world (virtual things), which are capable of being identified and integrated into communication networks [2]. These “physical things” can be vehicles, home appliances, and/or other items/devices embedded with electronics, software, sensors, and actuators. The ITU has also proffered a technical definition for the IoT as “a global infrastructure for the information society, enabling advanced services by interconnecting physical and virtual things based on existing and evolving interoperable information and communication technologies” [2]. Following these definitions, it is evident that communication networks are undoubtedly a fundamental component of any IoT reference model. Consequently, many IoT-based communication networks require that certain demands are satisfied, such as to provide low power consumption rates, long-range transmission, low cost, high data rates, and high scalability for different IoT applications [3]. However, since most existing cellular communication technologies such as the 3G, 4G, and 5G cellular technologies do not satisfy many of these requirements, such as the provision of low power consumption rates, long single hop transmission ranges, and low device development/deployment cost; thus, it is pertinent that alternative solutions be provided. Therefore, low power wide area network (LPWAN) technologies have recently sufficed as suitable communication preferences for many IoT-based applications.

LPWANs are popular for a number of unique characteristics, some of which are: they provide low power consumption rates [4], simplified network topologies, low developmental and purchase cost of devices, long transmission ranges (distance), simple and scalable deployment schemes, thin infrastructure, small data frame sizes, albeit at low data rates [4,5]. These characteristics closely align with the aforementioned demands of many IoT applications, thereby serving to motivate the present drive towards the development and performance improvement of new and existing LPWAN technologies [6,7,8,9,10]. Although LPWANs may not necessarily satisfy all the communication requirements of all the different IoT-based application areas, such as the provision of high data rates, nevertheless, they are envisioned to complement other communication technologies towards guaranteeing the holistic success of all IoT-based applications. Consequently, it is envisaged that the business capacity for LPWANs will surge in the coming years based on an estimation of over 700 million IoT devices to be connected over LPWAN standards by 2021 [11]. In this regard, some notable application areas that aim to benefit from LPWAN technologies include, but are not limited to ocean monitoring, smart city systems, smart grid, smart metering, soil monitoring, home automation, smart industries, wild life survey, intelligent transport systems, and forest management. This myriad of different application areas emphasizes the importance of LPWAN in the overall realizable architecture of the IoT.

However, many LPWAN technologies are typically beleaguered by specific PHY layer challenges such as increased interference, limited data rates, and spectral inefficiency. These limitations are inherent based on the fact that most LPWANs are largely deployed in the presently congested industrial, scientific, and medical (ISM) bands. Thus, an approach towards addressing these challenges may require the innovative integration of cognitive radio (CR) technologies in LPWAN. CR refers to an intelligent radio system developed primarily to address issues pertaining to inefficient spectral utilization. It was conceived following the relatively recent unavailability of radio spectrum for the deployment of new wireless technologies. CR aims to take advantage of unused (free) bands towards improving the user’s perceived quality of service (QoS) over different communication networks. There are several innovative CR technologies presently under development, which may greatly complement both existing and new LPWAN technologies; thus, it is pertinent to present an overview of these efforts and vision as it relates to enhancing different IoT applications.

Thus, in this article, we discuss independently both LPWAN and CR features and technologies as a foundation for the potential integration of CR in LPWAN. We highlight the concept of CR enabled LPWAN systems, termed CR-LPWANs. The potentials of CR-LPWAN systems are discussed along with the documentation of specific research challenges, which may limit the realization of CR-LPWAN systems. It is hoped that these research directions will spur researchers and LPWAN developers alike towards developing future ideas that will drive the next generation of IoT devices.

Summarily, the present article contributes as follows:

1. It presents insights and a recent overview regarding the use of CR in LPWAN. In particular, the use of CR as a potential solution to some known LPWAN problems is considered.
2. In addition to discussing the concept of CR-LPWAN, the present article provides a framework for integrating CR and LPWAN modules into a possible functional unit. It describes the front-end of a generic CR-LPWAN system describing how the interlink of each module may contribute to the support of effective CR-LPWAN systems.
3. We identify specific challenges that may mitigate against the realization of functional CR-LPWAN systems. Specifically, new insights are provided into different research challenges that require the use of adaptive CR technologies in LPWAN transceivers.
4. An up-to-date information is provided pertaining to the development and standardization of LPWAN systems in general. These include the provision of a summary concerning the origin of different LPWAN technologies, and an update regarding the different standard organizations and special groups involved in LPWAN development. An updated list of different LPWAN technology developers is provided with focus on their potential/adoption of CR technology. The use of CR is also discussed with regards to the general IoT architecture and the different challenges in this direction. Essentially, this article provides insights and future research considerations with regards to the concept of CR in LPWAN technologies, which may interest readers aiming to explore this new research trend.

The rest of this article is structured as follows: related survey articles are discussed in Section 2. An overview of LPWAN is provided in Section 3, with a similar overview of CR presented in Section 4. Then, we discuss the concept of CR-LPWAN in Section 5 where we look at its general concept via a PHY-layer front-end model, network architecture and support protocols. Section 6 presents the potential benefits of CR-LPWAN for IoT, whereas Section 7 discusses challenges and future research directions. We conclude in Section 8.

## 2. Related Works

In this section, we highlight the uniqueness of the present article by presenting evidence before this study of existing survey and literature review papers considered to be related to IoT and the role of CR and LPWAN systems in IoT-related applications. We found a number of survey articles pertaining to IoT and LPWANs, general overview of LPWAN, CR in specific IoT use-cases, and software-defined radio (SDR) in IoT applications. However, to the best of our knowledge, we found no survey article that synthesizes comprehensively information at the PHY, network, and protocol levels in order to interrelate the concept of LPWAN, IoT, and CR in view of their benefits, challenges, and future research directions. Other notable survey articles are discussed, for example, the survey on 5G networks for IoT in [12] discusses extensively about some IoT associated communication technologies. This survey provides insights into the 3rd Generation Partnership Project (3GPP)-based LPWAN technologies with focus on supporting massive to critical IoT use-cases. In particular, authors provided relevant state-of-the-art information pertaining to LPWAN technologies, such as the enhanced machine-type communication (eMTC), extended coverage Global System for Mobile communication (GSM) IoT (EC-GSM-IoT), Narrowband IoT (NB-IoT), and other non-cellular LPWAN technologies including LoRa, Sigfox, and Ingenu-RPMA, to name but a few. They noted that there are several open research challenges needed to be resolved towards realizing effective IoT systems, such as developing effective control and management systems for IoT networks.

In [10], the authors provided an overview of LPWAN technologies with focus on LoRa and LoRaWAN testbeds. Essentially, they discussed the flaws and strengths of works published with regards to LoRa technology. This was achieved by comparing existing testbeds designed to simulate and test different LPWAN technologies and protocols. They noted that the network performance of LPWANs could be affected by interference from other networks, in addition to the logical and geographical layout of LPWAN networks. Similarly, authors in [13] reviewed the opportunities, challenges and future directions in LPWANs. Notably, they mentioned challenges related to the development of LPWAN technologies, including spectrum limitation, coexistence issues, mobility management and scalability.

Raza et al., in [4], provided an extensive overview of LPWANs with focus on the design goals and techniques exploited in different LPWAN technologies. They surveyed different LPWAN technologies and standards noting that these LPWAN technologies share similar approaches, thus experiencing similar limitations and challenges. It was noted in [4] that most LPWAN standards focus on the physical (PHY) and medium access control (MAC) layers with little attention to the upper layers, thereby stimulating the need for additional research solutions to upper-layer challenges. Many other survey articles have been identified in [14,15,16,17], which focus on describing enabling technologies for LPWANs, including specific technologies such as LoRa, Sigfox, and NB-IoT. Most of these articles have identified a number of challenges and future research directions towards improving LPWAN technologies.

The use of CR to improve the performance of IoT systems was examined in some other survey articles. For example, the use of CR in smart grid communication networks (SGCN) was surveyed in [18]. Specifically, a detailed comparison was provided between wired and wireless communication technologies with focus on the use of modern concepts such as CR, smart utility networks (SUNs) and the use of TV white spaces (TVWS) within different smart grid (SG) environments. Khan et al., in [19], surveyed the use of CR in IoT with focus on applications, architectures, spectrum related issues and future research directions. Interestingly, they highlighted some reasons why IoT requires the incorporation of CR, including the case for efficient spectrum utilization, intelligent decision making, efficient bandwidth allocation for IoT objects, and the alleviation of interference situations.

Kobo et al., in [20], surveyed the challenges and design requirements for developing software-defined wireless sensor networks (SDWSN) with focus on their application to IoT. They discussed the use of CR in SDWSN and emphasized the importance of CR and the need for further research with regards to improving the performance of CR-based SDWSN. In a separate survey, authors in [21] discussed issues surrounding the use of LPWAN as a possible communication access technology for cognitive radio sensor network (CRSN) with special use in smart grid infrastructures. They focused on the benefits of LPWAN in CR-SG networks. They mentioned a number of research gaps including challenges in the implementation design model and issues surrounding the use of LPWAN in CRSN-based SG systems. Further, a smart unified communication solution was proposed to improve smart grid systems while mitigating existing challenges.

Different from the above-mentioned articles, our present article focuses on providing a background pertaining to the integration of CR in LPWANs. Noting that our survey may be one of the earliest overviews in this regard, and indicating that the concept of CR-LPWAN systems in IoT is still at its infancy, this article will be beneficial to the budding researcher who seeks to explore possible research challenges within the CR-LPWAN field.

## 3. Low Power Wide Area Network

Low power wide area network (LPWAN) is defined by the Internet Engineering Task Force (IETF) as a class of wireless technologies with characteristics such as large coverage areas, low bandwidth, possibly very small packet and application layer data sizes and long battery life operation [22]. Following these characteristics, LPWANs are widely deployed in many IoT-based communication networks, thereby garnering huge research audiences across academia, industry, and in different business domains [4]. Consequently, based on the potential benefits of LPWAN, we present a brief exposition regarding up-to-date available LPWAN standards, technologies, and their challenges. A brief look into the origin of LPWAN systems is provided, which presents a background for the current developmental strategies within the LPWAN market space. We then discuss different LPWAN technologies, standards and a possible generic architecture for LPWAN operation.

### 3.1. Brief Origin of LPWAN

We present a timeline to highlight the progression of LPWAN technologies from earlier versions to the present state-of-the-art LPWAN technologies. Such information should help readers to understand and to appreciate the current growth and future trends in the LPWAN market. The AlarmNet technology suffices as one of the earliest technologies built by alarm device manufacturing company (ADEMCO) in the early 1990s based on a 900 MHz network in order to monitor alarm panels [23]. It sent a small amount of data and transmitted at very low data rates. It served as the bedrock for the migration of remote alarm systems to the 2nd generation technologies, which provided both data and voice services [23]. A technology similar to modern LPWANs was the advanced radio data information service (ARDIS), which was a wireless wide area network that provided relatively low-speed networks for sales automation, fleet tracking and other online transaction processes [24]. ARDIS was later acquired by Motorola Incorporation [24].

Although the above-mentioned technologies lacked some characteristics as compared to modern LPWANs, for example, improved energy efficiency, nevertheless, they offered the foundation for the development of the presently known LPWAN systems. Furthermore, the emergence of the Internet and its present widespread application areas/potentials necessitated the need to improve these technologies as seen today. Much of the resurgence of LPWAN technologies was led by Sigfox, which originated in 2009 [25]. They built the first modern LPWAN in France and their success rate spurred quite a buzz in the industry. Sigfox technologies came at a period when radio devices were declining in cost and their usage was becoming pervasive. They were soon followed by other LPWAN interest groups such as the Ingenu, LoRa Alliance, and even traditional cellular players rolling out NB-IoT devices [4,9,26]. Particularly, LoRa technology was initially developed by Cycleo Incorporation in Grenoble, France, but was later acquired by SEMTECH in 2012. An overview of the timeline of the origin of different LPWAN technologies, along with their developers is presented in Table 1. Following this timeline, it is easy to appreciate how the LPWAN market has progressed with potentials for further growth in the nearest future. In this regard, it is our belief that the future of LPWANs will entail the integration and development of CR-LPWAN systems. A number of LPWAN technologies are discussed next, whereas standardization bodies involved in regulating the interoperability of LPWAN technologies will be discussed in subsequent subsections.

### 3.2. LPWAN Technologies

In this section, different LPWAN technologies are discussed. In addition to providing a detailed list of technologies, we identify specific LPWAN technologies that support CR functionalities. This exposition further emphasizes the budding consideration for CR and the need to commence widespread discussion about the integration of CR in LPWAN. To examine the potential for CR deployment, we identify different LPWAN technologies and their respective bands of operation, their current support for CR, their use of multiple channels, and their use of adaptive threshold techniques in their respective transceiver modules. In this regard, only the Semtech SX1276 transceiver module developed by LoRa provides an open source access to its underlying chipset technology.

It is best to encourage open access opportunities to different LPWAN transceiver architectures in order to fast track research contributions pertaining to the integration of CR in LPWAN. Further discussion in this regard is provided in Section VI. However, while this section serves only as an overview of different state-of-the-art LPWAN technologies, nevertheless, additional details regarding the technical comparative analyses of these technologies can be found in [4,15,27] for the sake of the interested reader. A few highlights regarding a number of LPWAN technologies are presented as follows:

A. **Sigfox:** Sigfox may be considered as the first major proprietary LPWAN provider in France [13]. Since its inception, Sigfox has proceeded in partnership with other network operators to ensure end-to-end LPWAN connectivity. They connect end devices to base stations via binary phase shift keying (BPSK) modulation on an ultra narrow bandwidth [4]. Sigfox technologies also provide CR capabilities, particularly in their base station infrastructures via SDR technology, which allows network and computing complexities to be managed via Cloud technology. Sigfox is quite popular with potentials to remain a strong competitor in the LPWAN market.
B. **LoRa:** Long Range (LoRa) is a LPWAN PHY layer technology developed by Semtech Corporation. Owing to its rich patronage by a wide range of researchers, LoRa easily seems to be the most popular and trending LPWAN access technology in the IoT market [4,28]. LoRa may also be popular because of its open standard communication protocol called LoRaWAN, developed by LoRa Alliance. LoRa adopts a spread spectrum technique, which allows for an increase in its scalability and data rate. Specifically, it is based on a chirp spread spectrum (CSS) modulation scheme integrated with forward error correction (FEC) codes, which allows for longer communication range as against using the frequency shift keying (FSK) technique [29]. LoRa devices are also capable of adjusting their transmission power to meet the regulatory requirements. LoRaWAN protocol is based on the ALOHA scheme [9]. It provides a fixed channel bandwidth of either 125 or 500 kHz in its uplink channels, and 500 kHz in its downlink channels [30]. LoRa is presently deployed by other notable developers such as Symphony Link^TM^ and LoRaBlink [31].
C. **Ingenu RPMA:** Ingenu was formerly known as On-Ramp Wireless [4]. It is a proprietary LPWAN technology designed to function strictly in the 2.4 GHz ISM band. It adopts a PHY layer technology based on random phase multiple access (RPMA) direct sequence spread spectrum (DSSS). RPMA enables multiple transmitters to share a single time slot. Ingenu-based RPMA achieves a high sensitivity level of about −142 dBm and a link budget of 168 dB [4,9,29]. It complies with the IEEE 802.15.4 k standard.
D. **Telensa:** Telensa is a LPWAN proprietary technology provider that renders end-to-end device connectivity [6]. It operates in the unlicensed sub-GHz ISM bands using a proprietary UNB modulation technique. Telensa complies with the European Telecommunications Standards Institute (ETSI) low throughput network (LTN) standard, which enables heterogeneous integration with other LPWAN technologies [4]. Telensa is widely considered for smart city applications such as intelligent traffic control and smart parking.
E. **Qowisio:** Qowisio is a proprietary LPWAN technology that combines its vertical stack with the use of LoRa PHY layer technology [4]. It ensures the provision of LPWAN services to end-users, with integration to cloud services for network operation. It adopts a proprietary UNB technology that provides most LPWAN characteristics such as low data rate and long-range transmissions.
F. **IQRF:** IQRF is a proprietary LPWAN technology developed by IORF Alliance in Pisek, Czech Republic [6]. Unlike other technologies, it uses mesh network topology supporting up to 239 devices with a single coordinator. It implements a dual communication mode to enable single or multimode peer-to-peer communication [13]. IQRF uses its own IQMESH protocol to communicate based on the mesh topology. It works in the unlicensed sub-GHz bands using 62 channels of 100 kHz bandwidth.
G. **LTE-M:** Long-Term Evolution, Category M1 (LTE-M) is proposed by the 3GPP group to provide connectivity for IoT devices. It can work in either full or half-duplex modes and provides a large receiver bandwidth of 1.4 MHz, which makes it relatively faster with a higher data rate (up to 1 Mbit/s in both uplink and downlink channels) than most other products [32]. It uses a deep sleep mode under the power saving mode (PSM) scheme and wakes only periodically to guarantee long battery lifetime. Its downlink channels work using both orthogonal frequency division multiple access (OFDMA) and 16-QAM modulation techniques, whereas its uplink employs the single carrier frequency division multiple access (SC-FDMA) and 16-QAM modulation techniques [33]. It aims to profit from the existing cellular infrastructure of the 3GPP group.
H. **NB-IoT:** Narrowband IoT (NB-IoT) is another scheme proposed by the 3GPP group for the connectivity of IoT devices. It supports up to 50,000 devices per cell using between 180 and 200 kHz bandwidth. It can operate in three different modes namely, the stand-alone, guard band, and in-band operating modes. Further details about these communication modes can be found in [34]. It uses quadrature phase shift keying (QPSK) modulation scheme for transmission in the licensed LTE frequency bands. It attains a maximum data rate of 200 kbps on half duplex mode. It complies with the 3GPP standardization specifications.
I. **Weightless:** Weightless is an open-standard LPWAN technology that operates in the sub-GHz unlicensed spectrum [35]. It achieves this feat via three different versions namely, Weightless-W, Weightless-N, and Weightless-P. Weightless-W leverages white space CR technology via dynamic spectrum access. However, it suffers from a shorter battery lifetime as against the other two versions [35]. Weightless-N operates in the unlicensed spectrum using narrow band protocols developed by NWave. Weightless-P, on the other hand works on a fully bidirectional based communication protocol developed by M2COMM’s Platanus technology [35].
J. **Adaptrum:** Adaptrum is a relatively newer technology compared to other known brands such as LoRa, Sigfox, and Weightless. There are few investigations available at the moment concerning the technical comparative characteristics of Adaptrum against other LPWAN technologies owing to its proprietary standards; nevertheless, it is considered to be a rare class of LPWAN technologies that claim to support TV white space usage via CR technology [36].
K. **Nwave:** Nwave is a LPWAN technology often used interchangeably with Weightless technologies [37]. Nevertheless, they differ, particularly in that Weightless developers use Nwave technologies to guarantee the use of TV white spaces [38]. Although Nwave technologies are known to provide Internet facilities, nevertheless, in recent times, they have been enabled for wireless IoT services and technologies in cities, rural areas, and remote areas [39]. Technically, Nwave technologies are tightly interlinked with Weightless technologies, thereby making it difficult to differentiate them.
L. **Platanus M2COMM:** Platanus is a wireless networking technology developed by M2COMM and considered to be a LPWAN technology because it provides ultra-low energy consumption rates with network coverage far longer in range than Wi-Fi, Bluetooth, Zigbee and BLE (i.e., from several meters to 10 km) [40]. Platanus is used in the Weightless-P technology to achieve fully bi-directional communication. Another LPWAN module developed by M2COMM is the Uplynx highly integrated system on a chip (SoC) module [41], which is deployed to simplify the development of LPWAN IoT applications. There is a very tight working relationship between Sigfox and M2COMM; consequently, both technologies are often used interchangeably in the literature; nevertheless, it is worth noting that they are distinct LPWAN technologies [12].
M. **Wi-SUN:** Wireless smart utility network (Wi-SUN) is an industry alliance that promotes interoperability between wireless standards in the IoT market. They work closely with the Internet Engineering Task Force (IETF) in order to ensure that Internet Protocol (IP) and transport layer protocols of LPWAN technologies are adequately standardized [25]. A prominent proponent of Wi-SUN is their field area network (FAN), which poses so many use-cases for IoT applications. They adopt the 6LoWPAN technology primarily for header compression [42]. Wi-SUN supports full IP frames with header compression in order to optimize bandwidth. They intend to maximize battery lifetime and guarantee long-range communication.
N. **Amber Wireless:** Amber Wireless GmBH is an electronics company that designs and manufactures wireless connectivity solutions. Although they may not be full-time players in the LPWAN market, nevertheless, they are known for low power products, particularly for shorter range transmission [14]. Their access modules are used primarily for home automation applications and smart metering.
O. **Starfish:** Starfish is a recent international wireless IPv6 network service deployed particularly for IoT applications. It is a technology developed by Silver Spring Networks [33]. Use-cases for Starfish technologies include intelligent traffic light control, wireless sensor networks applications including water, energy, traffic and safety monitoring [33]. Starfish uses a wide range of LPWAN standards-based communication protocols, particularly the IEEE 802.15.4 g. Although little may be known regarding the technicalities of Starfish technologies, nevertheless, Starfish projects are gaining wide spread use in many IoT applications.
P. **Symphony Link and Ensemble:** Symphony Link and Ensemble is a proprietary technology of Link Labs, which uses LoRa at the PHY layer and a different MAC architecture to provide proprietary services [43]. It uses an eight-channel base station that operates in the 433 MHz or 915 MHz ISM bands, and in the 868 MHz band in Europe. It covers a transmission range of over 16 km over a back-haul based on Wi-Fi or cellular network [43].

The entire LPWAN technologies listed above are appraised in Table 2 and ranked according to their supposed integration of CR in their respective LPWAN technologies. However, the Adaptrum and Platanus technologies were omitted for a lack of detailed technical information from the proprietary developers regarding the parameters of comparison in Table 2. Ranking was conducted based on the number of ticks in Table 2, which aims at assessing the use of specific CR technologies and strategies in the different LPWAN technologies. Following Table 2, it is noted that both Nwave and Weightless adopt the most number of CR techniques and strategies in their respective technologies, which agrees with similar claims in the literature that they presently support CR in their different technologies [37].

### 3.3. LPWAN Standards

Based on the growing list of LPWAN technologies, it is evident that there is considerable diversity among LPWAN technologies, which breeds a number of issues that may stifle their growth and interoperability. For example, while some developers are contending the introduction of IPv6 in their LPWAN technologies, Wi-SUN on the other hand has commenced support of IPv6 using the 6LoWPAN technology [25]. These uncoordinated diversities may affect the end users negatively such that migration between different technologies will become greatly hampered. This may ultimately restrict the widespread acceptance of LPWAN technologies. Consequently, some standards have been developed to articulate the different concerns pertaining to the interoperation of LPWAN technologies. However, these standards are incoherent because of a lack of a unified front amongst LPWAN developers. Thus, a number of international bodies are presently involved in the standardization process of LPWAN technologies, for example, the IETF has established a new LPWAN working group (WG) to address IPv6 issues over LPWANs, and to establish how LPWANs will adapt into a mutually beneficial ecosystem [25].

In this regard, there are two different assemblages working to standardize the LPWAN ecosystem. These are the standard development organizations (SDOs) and the Special Interest Groups (SIGs) [4]. Notwithstanding, there are other consortiums working and advocating for the interoperability and standardization of different IoT components. For example, the Industrial Internet Consortium (IIC) aims to set up an architectural framework for industrial IoT by coordinating connected objects that use common architectures, interoperability, and open standards. Most prominently, the SDOs and SIGs are widely known as proponents for the interoperability of different IoT standards. The SDOs comprise known organizations such as the European Telecommunications Standard Institute (ETSI), the Third Generation Partnership Project (3GPP), Institute of Electrical and Electronics Engineers (IEEE), and the Internet Engineering Task Force (IETF) [4]. An overview of these SDOs based on their area of focus, membership, and membership count is provided in Table 3. The SIGs on the other hand comprise individual industrial alliances such as the LoRa Alliance, Weightless-SIG, and the DASH7 Alliance [4]. We provide an overview of the developments made thus far with regards to the standardization of LPWAN technologies.

#### 3.3.1. Standard Development Organizations (SDOs)

A few notable SDOs are discussed as follows:

##### IEEE Standards

The IEEE has established two different standards, namely, 802.15.4 and 802.11 standards, designed to ensure range extension and reduction in the power consumption rates of LPWANs. Under the 802.15.4 standard lies the IEEE 802.15.4 k task group (TG) and the 802.15.4 g TG [44]. The 802.15.4 k option is designed for low energy critical infrastructure monitoring (LECIM) applications intended to operate in the ISM bands (sub-GHz and 2.4 GHz bands) [44]. They propose to use DSSS and FSK modulation techniques at the PHY layer to increase range and node scalability [45]. The MAC layer supports carrier sense multiple access with collision avoidance (CSMA/CA) and ALOHA with priority channel access (PCA) [28,46]. The 802.15.4 k specifies power levels of about −130 dBm for base stations [47]. A transmitter power of 15 dBm is proposed in the 433 MHz spectrum. One technology currently complying with this standard is the Ingenu LPWAN technology [28,46]. The IEEE 802.15.4 g standard is considered for low data rate wireless smart metering utility networks. This TG proposes to use FSK, OFDMA, and QPSK to improve data rate. Similar to 802.15.4 k, the IEEE 802.15.4 g standard is specified for use in the ISM (sub-GHz and 2.4 GHz) bands.

The 802.11 wireless local area network (WLAN) standard is also considered for IoT applications. In particular, the IEEE 802.11 ah is designed to reduce power consumption rates and to extend the range of long-range low power (LRLP) systems [4,14]. This standard proposes LPWAN operation in the ISM sub-GHz bands with an intention to provide longer range and lower power consumption rates as against other known WLAN standards such as ZigBee and Bluetooth. Consequently, the 802.11 ah is a favourable technology for IoT applications since it provides low power consumption rates comparable to Bluetooth with an added benefit of higher data rates and wider coverage range.

##### European Telecommunications Standard Institute (ETSI)

The ETSI is saddled with establishing bidirectional communication protocols in LPWAN technologies [4,28]. They are striving to reduce the electromagnetic radiation levels of LPWAN technologies by reducing their payload sizes and ensuring transmission at low data rates. They propose different interfaces and protocols to support communication between end devices, base stations and network servers [48]. The ETSI refers to its LPWAN standard as low throughput networks (LTNs) [4,28]. The LTN standard does not restrict LPWAN technologies to any particular modulation or access technique. Nevertheless, it supports provision for either ultra narrow band (UNB) or orthogonal sequence spread spectrum (OSSS) methods. With regards to IoT-based applications, the ETSI has published some standards including the standard on the technical characteristics for UNB short range devices (SRD), which operates in the UHF spectrum below 1 GHz, the standard on use-cases and system characteristics for LTN, and the standard on IPv6-based IoT. Some LPWAN developers such as Sigfox, Telensa, and Semtech are presently involved with ETSI in order to standardize their respective technologies.

##### Third Generation Partnership Project (3GPP)

The 3GPP group is presently evolving its technologies to ensure use for IoT applications [4,13,28,49]. They have stripped their cellular standards of unnecessary complexities, reduced costs, extended transmission range, and ensured prolonged battery lifetime. LTE technologies are being enhanced particularly for narrowband (NB)-IoT applications [50]. NB-IoTs are designed to provide low cost, long coverage, low data rates and low power consumption rates to satisfy LPWAN requirements. For example, in order to extend battery lifetime, the 3GPP are planning to deploy either PSM or extended discontinuous reception (eDRx) schemes [4,12,13]. Both methods use deep sleep mode operation for end devices to provide longer hours or even days of network activities. The NB-IoT is a narrow band technology with low bandwidths of 180 kHz with the possibility to serve as many as fifty thousand end devices in a particular cell [4]. It uses frequency division multiple access (FDMA) and OFDMA in the uplink and downlink channels, respectively. With an established infrastructure based on older 3GPP cellular technologies, NB-IoT is expected to be a strong competitor in the IoT market in the coming years.

##### Internet Engineering Task Force (IETF)

The IETF strives to standardize areas related to IP-based connectivity of LPWAN systems [25,51,52]. It is an open standard organization with no formal membership or membership requirements. All participants and managers are volunteers with funding provided by employers or sponsors. The IETF presently considers IPv6 particularly for low power wireless personal area networks (6LoWPAN) [53,54]. One requirement is to ensure that the IP stack is lightweight enough to guarantee strict compliance with the limitations of LPWAN technologies, such as their operation under strict regulations in ISM bands, the asymmetry of their uplink and downlink channels, and the existence of different packet formats from different LPWAN developers in the IoT market [25,51,52]. Following the completion of the task of the 6LowPAN IETF IoT WG in 2014, another WG called the 6Lo WG was initiated in order to cover a wider range of radio technologies including Bluetooth, cordless phones, and Digital Enhanced Cordless Telecommunication (DECT) devices. The IETF group aims to solve issues of header compression, fragmentation and reassembly, management, and security, integrity and privacy issues [4].

#### 3.3.2. Special Interest Groups (SIGs)

This section updates the discussion on SIGs as presented in [4]. We include newer alliances such as the IQRF Alliance and the Wi-SUN Alliance. We summarize these SIGs in Table 4 according to their respective areas of focus and constituting members. We also examine whether they are inclined towards integrating CR technologies or not. They are discussed as follows:

##### LoRa Alliance

LoRa Alliance is an industry-based standard currently promoting LoRa proprietary PHY layer technologies for LPWAN connectivity [4,55]. It is an open, non-profit association of members with a mission to standardize LPWAN on a global scale for IoT applications. It strives to ensure the success of its proprietary LoRaWAN technology by sharing knowledge and experience required for interoperability between operators. It was launched in April, 2015. The alliance adopts ALOHA scheme at the MAC layer and spread spectrum technologies at the PHY layer to accommodate additional end-users [49]. It establishes different classes of users such as the Class A devices that require the longest lifetime with the highest latency, the Class B devices that can schedule downlink receptions from base stations at certain time intervals, and the Class C devices that are mains-powered to continuously listen and receive downlink transmissions. There are a number of technical documents, which detail the development of LoRa and LoRaWAN technologies/standards, some of which can be found in [4,9,10,56,57].

##### Weightless-SIG

The Weightless-SIG group is a non-profit, global, and member-based organization geared towards developing open standards for IoT connectivity. It was formed in 2012 particularly to coordinate activities surrounding the delivery of standards for wide area IoT connectivity. It has proposed three standards, which operate in both the licensed and unlicensed spectrum [4]. These standards are:

1. Weightless-W standard, which uses TV white spaces based on different modulation schemes such as 16-quadrature amplitude modulation (16-QAM) and differential binary phase shift keying (D-BPSK) [58].
2. The Weightless-N standard, which is a UNB standard that focuses only on simplex communication mode. It provides for higher energy efficiency and lower device cost as against the Weightless-W.
3. The Weightless-P standard, which provides full duplex communication mode based on QPSK and Gaussian minimum shift keying (GMSK) modulation techniques. It provides a data rate between 0.2 and 100 kbps [4,58].

##### DASH7 Alliance

DASH7 is an industry alliance that employs narrow band modulation schemes domiciled in the sub-GHz bands [13]. It proposes a tree topology as against the star topology of the LoRa alliance. The DASH7 alliance embraces an always ON base station set-up to ensure constant network connectivity, which differentiates it from other standards [13].

##### IQRF Alliance

The IQRF Alliance is an open community of international IoT professionals that aims to deliver wireless IoT devices and solutions based on the IQRF technology [59]. A few examples of hardware devices developed under the IQRF Alliance include the industrial gateway for IQRF networks, the outdoor industrial gateway (IP68 rugged industrial gateway), and the network traffic analysis modules designed for IoT network analysis, to name but a few. Many IQRF Alliance-based technologies are often built on an integrated mesh networking scheme, which aims to provide low power consumption rates using deep sleep modes, and to provide enhanced security solutions based on the Advance Encryption Standard (AES) 128 encryption scheme [59]. In routing terms, the IQRF Alliance are known to advance the IQMESH routing protocol, which is aimed at providing reliable performance in harsh radio frequency (RF) environments [60].

##### Wi-SUN Alliance

Formed in 2011, the Wi-SUN Alliance comprises individuals who came together to form a global organization that drives the adoption of interoperable SUN [61]. The alliance focuses on applications including but not limited to energy conservation, automated metering control, and smart cities [62]. Technically, the Wi-SUN Alliance produces test equipment, network emulators, communication modules, and spectrum analyzers [61]. Some Wi-SUN solutions are based on LoRa using LoRa modulation spread spectrum scheme in the sub-GHz band.

##### IoT World Alliance

The IoT World Alliance formerly known as the M2M World Alliance consists of a global partnership of telecommunications providers striving to enable seamless IoT device connectivity around the world, particularly for their proprietary technologies [63]. The alliance provides a single global subscriber identity module (SIM) that works on all member networks, they provide centralized management of connected devices and a single web platform portal to manage connections worldwide [63]. The interest of IoT World Alliance may be more or less related to IoT marketing and education.

Other alliances, which are not fully discussed here are as follows, but not limited to the Hypercat Alliance, Allseen Alliance, Open Mobile Alliance, Internet Protocol for Smart Objects (IPSO) Alliance, Continua Alliance, Connected Lighting Alliance, ZWave Alliance, and the EnOcean Alliance. These alliances are geared towards the standardization and interoperability of all facets of the IoT. This ever-growing list of alliances emphasizes the intense focus on IoT development and on the development of improved LPWAN technologies.

In summary, Table 5 provides a list of consortia involved in the standardization of different IoT technologies. Essentially, in the absence of an all-encompassing alliance, the IoT market will continue to comprise different non-interoperable technologies, which may stifle the expected growth of the LPWAN market.

### 3.4. General LPWAN Architecture

Owing to the different LPWAN technologies available today, it is difficult to present an all-encompassing architecture that unifies all LPWAN technologies. Such an inability to generalize may be obvious since each developer provides a unique architecture for its devices. One such popular LPWAN architecture is the star-of-stars network architecture, which is an architecture widely deployed in both Sigfox and LoRa-based infrastructures [64]. In this section, we identify specific commonalities between different LPWAN architectures in order to describe a generalized LPWAN architecture. This will provide LPWAN developers with an architecture that spurs interoperability.

There are several specific architectures identified in the LPWAN literature. For example, a LoRaWAN network architecture was presented in [65] wherein end nodes (IoT devices) typically connect to different gateways/concentrators via LoRa RF and LoRaMAC. The gateway (GW) connects to a network server (NS) via 3G/4G/Ethernet/Fibre back-haul, whereas the NS connects to application servers (AS) via TCP/IP Secured Sockets Layers (SSL) payloads. In another article [66], authors presented a more detailed LPWAN architecture called OpenChirp. The OpenChirp network was built using LoRaWAN, which allows users to register devices, describe transducer properties, transfer data and retrieve historical values. Unlike the architecture in [65], OpenChirp connects its GWs through a publish-subscriber server that decouples the producers and consumers of information according to their timing and availability conditions. LPWAN servers and serializers are both classified within the service domain in the OpenChirp architecture, and they connect to application services through a Hypertext Transfer Protocol (HTTP) server.

Similar to [65], authors in [67] presented a typical LoRaWAN network architecture, which comprises four basic sectors involving the connection of end devices to the GW, and then to the Internet before arriving at the NS. In a survey article, Lavric and Popa [16] highlighted a LoRa architecture wherein end devices situated in different cloud clusters would connect to the LoRaWAN gateway through a LoRa medium, whereas the GW connects to an NS via an IP back-haul. A Sigfox network architecture was described in [37] in which objects comprising client applicative boards and Sigfox MODEMs were connected to Sigfox base stations via radio links. These Sigfox base stations were in turn connected to a Sigfox cloud service prior to arriving at the client site. Thus, most LPWAN architectures, ranging from LoRaWAN to Sigfox architectures, commonly comprise end nodes that connect to a GW, then to an NS prior to arriving at an application server.

**Figure 1:**
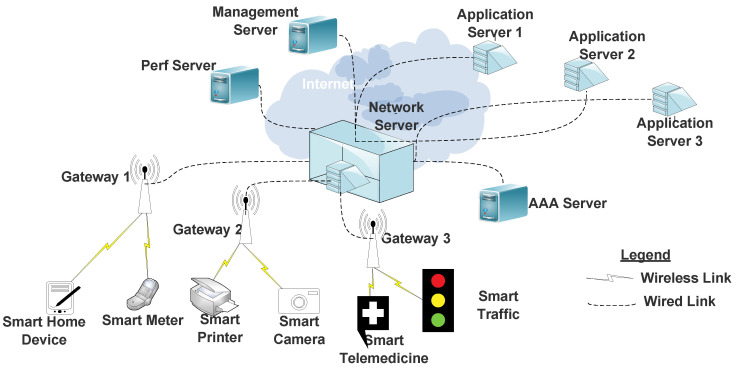
A simple LPWAN architecture.

Following the above, a simple LPWAN architecture is presented in Figure 1. In this general architecture, all IoT devices (including wireless sensor network (WSN) devices) would connect to the GW via LPWAN access technologies. These access technologies can be deployed through specific providers including LoRaWAN, Sigfox, Wi-SUN, or NB-IoT access providers. The GWs aggregate multiple IoT messages and send them to the NS, which is located on the internet or can be accessed through some IP technology either through wired or wireless connectivity. The NS aggregates data messages from the different GWs for onward transmission to an application server. At the NS, different services can be provided, including security services (authentication) through the AAA server, performance evaluation through the Perf server, and management processes through the management server.

In terms of developing a generic architecture, the IETF is at the forefront towards the realization of a seamless interoperability platform for both new and existing LPWAN technologies. Thus, one major task of the IETF WG is to identify common functionalities in the LPWAN-GW and to standardize new and existing protocols required for seamless interoperability [25]. Such a generic architecture should ensure that LPWAN characteristics such as long battery lifetime, low data rates, low deployment/device costs, and scalability, are maintained across heterogeneous LPWAN technologies. Thus, the goal of a generic LPWAN architecture is to converge diverse radio technologies towards a central commonality for easy migration between different technologies. This easy migration will ultimately benefit end-users, which should be the case for the success of any new technology.

## 4. Cognitive Radio

In this section, we briefly introduce and discuss the concept of CR towards presenting the budding researcher with a synopsis of what CR entails, its specific functions, and its possible network concepts.

### 4.1. Brief Background

The study of CR is presently a popular and interesting research area because it attempts to address the problem of spectrum underutilization. Over the past 20 years, the wireless communication industry has experienced a surge in the number of new wireless technologies deployed to improve the QoS of end-users [68]. Each newly deployed wireless technology demands new and unique frequencies to be allocated for its operation (i.e., fixed spectrum assignment). However, owing to the dwindling availability of usable RF bands, particularly across the very high frequency/ultra high frequency (VHF/UHF) bands up until the microwave band, it has become obvious to most national frequency managers worldwide that spectrum is becoming scarce (spectrum scarcity). Such a reality of spectrum scarcity poses a significant threat to the growth of the wireless communication industry [69]. However, several research findings have revealed that most allocated frequency bands are often underutilized as opposed to being scarce, with utility levels ranging between 15 and 85% [70]. Thus, the concept of CR was conceived supported by the concurrent advances in the development of SDR technologies [71]. Consequently, the study, development, and deployment of CR technologies has since grown exponentially in the literature following its potential use of free spectra for opportunistic communication.

Technically, CR is defined by the Federal Communications Commission (FCC) as: “A radio or system that senses its operational electromagnetic environment and can dynamically and autonomously adjust its radio operating parameters to modify system operation, such as maximize throughput, mitigate interference, facilitate interoperability, access secondary markets” [68]. This definition highlights some specific CR characteristics as follows: CR technologies must provide adaptive and autonomous spectral awareness (spectrum sensing (SS)), detect available channels (spectrum decision making), dynamically adjust its radio operating parameters (spectrum mobility), and conduct concurrent communication (spectrum sharing). In the next subsection, we present only a brief overview of these characteristics.

### 4.2. Cognitive Radio Functions

**Figure 2:**
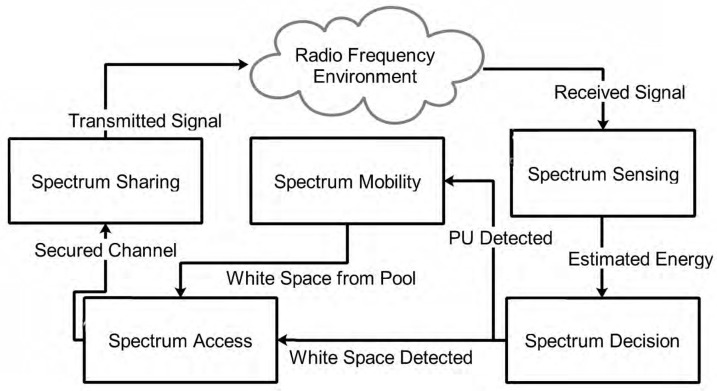
The cognitive radio system.

A general overview of the different functions in a typical CR system (CRS) is shown in Figure 2. There are five basic functions of a CRS described as follows:

1. **Spectrum Sensing (SS):** A typical CR device is equipped with a radio front-end to scan (sense) its immediate electromagnetic environment for the presence/absence of primary user (PU) signals. In this case, PU refers to the licensed owner of the spectrum. There are different approaches proposed for SS, including the use of the energy detector (ED), matched filter, cyclostationary detector (CD), eigenvalue method, covariance method, and prediction-based approaches [72]. Another method currently deployed for spectral awareness is the geolocation database approach [28,73]. In this case, a central database comprising geographic coordinates and the respective RF signal distributions in such environments is established. Consequently, CR devices would connect to these databases in order to acquire PU information before decision is made.
2. **Spectrum Decision Making:** Following the measurement of the energy content of a specified band, the CR device progresses to the spectrum decision-making phase wherein decision is made concerning the presence/absence of PU signals in the band. In the case where only the signal’s energy values are known, then decision is made based on whether the received energy values exceed a specified threshold value or not. However, in cases where certain characteristics about the PU signal are known a priori, for example, the cyclic frequency, then the CD technique can be deployed, albeit at the expense of long processing times.
3. **Spectrum Access:** Spectrum access entails the use of information pertaining to the absence of PU signals in a band (white space) by carefully designed CR MAC protocols in order to adjust transmission parameters. This ensures that the new operating radio spectral can be conveniently and efficiently used for opportunistic communication.
4. **Spectrum Mobility:** Information regarding the presence of PU signals in a band (black space) is used to ensure hand-off/change of transmission parameters in order to prevent interference to PU operators. This ensures seamless communication between CR devices in the new white spaces.
5. **Spectrum Sharing:** Spectrum sharing guarantees effective communication between CR devices and coexistence with PU transceivers without inflicting harmful interference. This is achieved by specific protocols designed to operate below a predefined interference level.

### 4.3. Cognitive Radio Network

CR network (CRN) is a network that comprises more than one CR device equipped with cognitive capability and reconfigurability, which intercommunicate by changing their transmitter parameters based on their respective interaction with the radio operating environment [74]. There are three different CRN schemes namely: Interweave CRN, Underlay CRN, and Overlay CRN, briefly discussed as follows:

1. **The Interweave CRN:** The Interweave CRN enables a CR device to use spectrum only if no PU is active. Thus, PU activities are constantly being monitored to prevent interference from CR transmissions.
2. **The Underlay CRN:** In the Underlay CRN, CR devices transmit with low power in the presence of PU activities. However, CR power is strictly ensured below a predefined noise/temperature level to prevent interference [75]. Ultra wide band communication approaches are typically used in Underlay CRN scheme.
3. **The Overlay CRN:** In the Overlay CRN, CR devices use code books and messages to identify the PU, and then mitigate interference by relaying their messages based on a difference code book [75]. The CR power level is typically not an issue of concern in the Overlay scheme.

CRNs are indeed to be beneficial to the development of IoT in general, and towards enhancing the performance of LPWAN technologies. Consequently, in subsequent sections, we shall discuss the burgeoning concept of CR in LPWAN.

## 5. Integrating Cognitive Radio in LPWAN for the Internet of Things

Developing effective and efficient IoT devices/applications comes with its unique challenges, which may be solved by leveraging the advantages of CR technology. We motivate the need to integrate CR in LPWAN based on extensive views, opinions, and efforts in the literature. We then discuss CR-LPWAN at the PHY layer based on a simple front-end model, and then a typical network architecture is discussed to support CR-LPWAN systems.

### 5.1. Motivation for CR-LPWAN

Compared to other facets of LPWAN development, such as designing better protocols, modulation techniques, and improved access technologies, the current literature provides little on the use of CR in LPWAN. Nevertheless, a few proprietary technologies, such as Sigfox, have led the way in integrating CR in their designs. Sigfox deploys CR technology in their base stations and connects them to back end servers via an IP network [12,19,28,34]. For proprietary reasons, little or nothing is known regarding the type of CR technology deployed; nevertheless, Sigfox technologies are known to operate in both the unlicensed ISM bands, including 868 MHz in Europe, 915 MHz in North America, and 433 MHz in Asia, and in the licensed band, which is made possible most likely by the use of CR capabilities. This major step has also been considered by the Weightless Alliance in their Weightless-W technology [5], thereby further enriching the drive for the widespread development and deployment of CR-LPWAN technologies.

Regarding why CR should be deployed in LPWAN, authors in [5] noted that because of strict regulations pertaining to the use of limited maximum duty cycle rates in the ISM bands, and its confining effects on the capacity of IoT networks, it may be worthwhile to consider the inclusion of CR in LPWAN. Even though some LPWAN standards, such as the LoRaWAN are yet to incorporate CR in their specifications, authors in [5] however argued that incorporating CR in LPWAN will present several advantages including improved spectral utilization, less transmission power constraints, possibility for longer transmission ranges, and lower device cost [5]. Moon in [42] emphasized the need to incorporate CR in LPWAN in order to maximize spectrum capacity available in the licensed bands while minimizing interference to the licensed network. In this regard, a dynamic spectrum access strategy for CR-LPWANs, which operates both in the licensed and unlicensed bands was proposed. Moon [42] demonstrated that spectrum capacity was maximized and strict QoS of licensed users were maintained.

It was noted in [12] that CR is a key network enabler for 5G mobile networks including the IoT network. This was attributed to the need to maximize spectral resources in order to support increasing and higher demands for new emerging IoT applications. It was further noted that CR can be incorporated by introducing a spectrum coordinator in the non-access stratum, which will enable cellular technologies to dynamically lease spectrum for IoT applications. These observations have strengthened the need to develop CR-LPWAN technologies.

### 5.2. CR-LPWAN at the PHY Layer

Having motivated the need to develop and deploy CR-LPWAN technologies, a simplified concept of a PHY-layer front-end model of a CR-LPWAN system is presented. Recall that there are different available LPWAN technologies mentioned (see Section 3), which makes rendering an all-encompassing model a difficult task. Nevertheless, the model to be described is considered following ideas gleaned from three different well known platforms, including LoRa, Sigfox, and the Ingenu architectures.

**Figure 3:**
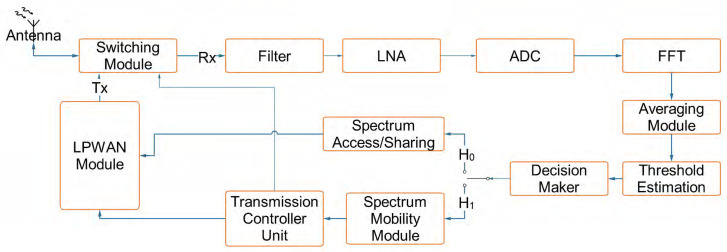
A simple CR-LPWAN PHY layer architecture (adapted from [84]).

In this regard, such a simple CR-LPWAN PHY layer architecture is presented in Figure 3 wherein the surrounding spectrum is first scanned by the CR-LPWAN system in order to determine whether or not white spaces are available for use. To achieve this, the received signal is filtered to the required bandwidth of interest and fed to a low noise amplifier (LNA) to reduce noise samples. These signals are converted to their corresponding digital form and a fast Fourier transformation (FFT) operation is performed. The averaged signals are passed to the threshold estimator to determine a suitable threshold value for signal detection. Decision is made regarding the presence ($H_{1})$ or absence ($H_{0})$ of PU signals in the band. If the outcome is $H_{0}$, then the spectrum access/sharing module is engaged, which formats the signal as required by the LPWAN module. However, if the outcome is $H_{1}$, the spectrum mobility module is activated for expedite withdrawal from the band. This command is fed to the transmission controller, which disengages any on-going transmission process in the LPWAN module and simultaneously activates the switching module to begin sensing (receiving mode) rather than transmitting. However, if the $H_{0}$ subsists, then the LPWAN module is activated for transmission. Some details pertaining to the individual blocks of this model are highlighted as follows [76]:

1. **Antenna:** Antenna design plays an important role in improving the radio performance of CR-LPWAN systems. Effective antenna design can be vital in ensuring proper propagation characteristics and also in conserving energy consumption rates. Essentially, antenna wavelengths must be made to match the operating frequency of the device. The antenna form factor used also determines to a large extent the gain (or loss) of the system, in addition to the gain in the directivity of the antenna. Low cost antenna technologies were discussed in [77] with particular focus on cost efficient antennas for 868 MHz band. The antenna design in [77] comprised of an inverted F antenna (IFA) topology with simulation results of about -6dB reflection coefficient in the 850–893 MHz band. A dipole radiation pattern was proposed. These are characteristics that should be considered in the antenna design of any CR-LPWAN system. Lizzi et al., in [78] discussed the design of miniature antennas for IoT applications. Similar to [77], Lizzi’s design [78] adopts the IFA topology. He noted that the overall IFA length is responsible for the lower antenna resonance relished in LoRa communication systems [78]. Essentially, proper consideration must be given to antenna design and structure for the efficient and effective deployment of CR-LPWAN systems.
2. **Switching Module**: The switching module is a Duplexer that enables bi-directional transmission over a single path. Its function is to separate the receiving path from the transmitting path while ensuring that a common antenna is shared. We suggest that CR-LPWAN models should deploy a switching module, whereas the choice of whether or not half or full duplex mode should be used, can be an application-specific decision.
3. **Low Noise Amplifier (LNA):** The LNA amplifies the received RF signal to increase the signal to noise ratio (SNR) at the CR-LPWAN receiver. We identity a few low-cost LNA modules that can be in CR-LPWAN systems. The duplex current reused CMOS LNA is a notable example with complementary derivative superposition technique that can be used in IoT devices [79]. We suggest that LNAs be deployed to ensure that low power consumption rates are maintained to maximize the battery lifetime of CR-LPWAN systems.
4. **Filtering and Down Conversion**: The front-end of the CR-LPWAN system filters and down-converts the signal frequencies to their intermediate frequencies (IF). It achieves this by using a mixer to obtain the in-phase and quadrature signal components at the IF. It can then process the signal either at both the IF and baseband levels to minimize design complexities in CR-LPWAN systems.
5. **Analogue to Digital Converter**: The ADC transforms the analogue signal to its digital form. As an example, CR-LPWAN systems can be deployed with sigma delta ADCs to convert the input data and then all subsequent signal processing and demodulation processes can be performed in the digital domain.
6. **Fast Fourier Transform Module**: CR-LPWAN systems will adopt an FFT module to compute the signal’s input energy. For example, an FFT LogiCORE IP core module can be used in this regard since it implements the Cooley-Tukey FFT algorithm in an efficient manner [80].
7. **Threshold Estimator:** Most CR-LPWAN systems will be required to compute threshold values for accurate signal detection. Since this process often depends on the noise floor, simple threshold estimation techniques can be considered in CR-LPWAN systems such as the fixed threshold technique. However, while the fixed threshold technique is readily deployed in most LPWAN systems, e.g., LoRa [66,81,82], other approaches can be used such as the peak and average threshold mode techniques. In the peak threshold mode, the threshold level corresponds to the peak value of the received signal strength (RSS). In the absence of an input signal, or during the reception of zero bits, the acquired peak value is decremented until it reaches the noise floor threshold [83]. On the other hand, the average threshold mode simply computes the mean of the entire dataset supplied by the RSS block. However, this approach may not be efficient in the presence of DC (direct current) encoded data. Summarily, it is worth noting that appropriate configuration of threshold values is fundamental to the success of CR-LPWAN systems, and its choice may be application dependent.

**Figure 4:**
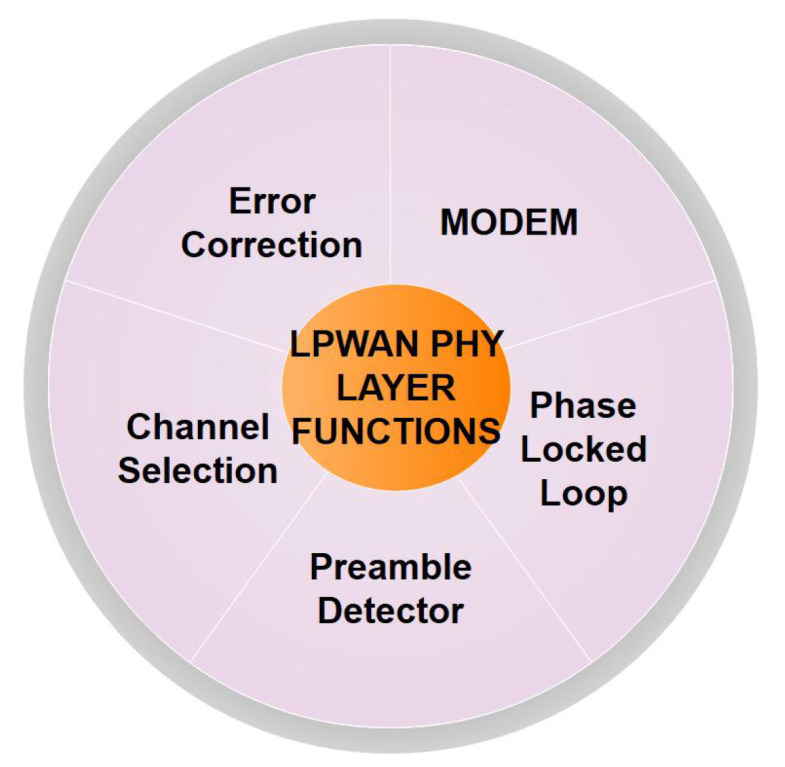
Essential PHY layer functions to be executed in the LPWAN module of the front-end model.

The decision module refers to a comparator, which can be used to determine the presence or absence of PU signals in the input band. Other functions such as modulation/demodulation are applicable based on existing brands such as LoRa, Sigfox, or Weightless. Summarily, the somewhat generic framework of Figure 3 can be leveraged to develop CR-LPWAN systems alongside an appropriate choice of the CRN scheme. Other PHY layer functions that can be executed within the LPWAN framework in the CR-LPWAN system are shown in Figure 4. These are known functions, which are often developed for use in most proprietary LPWAN technologies, for example, in the SX1276 LoRa architecture [83].

### 5.3. Network Architecture to Support CR-LPWAN

**Figure 5:**
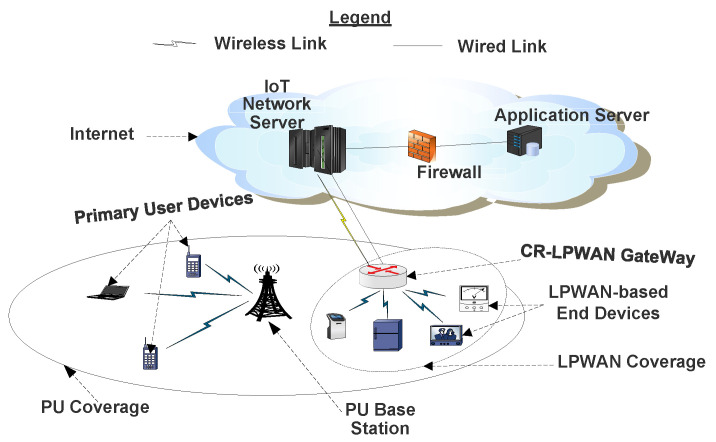
A simple CR-LPWAN-based network architecture (adapted from [84]).

A simple network architecture to support CR-LPWAN systems is depicted in Figure 5. Although different architectures may be required for different IoT use-cases, nevertheless, the architecture of Figure 5 is only presented to demonstrate the possibility for CR-LPWAN. Typically, CR-LPWAN devices for different IoT applications can be situated or located within a larger PU network (often cellular networks). The CR-LPWAN GW senses for when such PU signals are absent over a period of time in order to initiate opportunistic communication. They will continue to sense for re-initiated PU signals in order to quickly vacate such occupied bands.

In this regard, there are three possible schemes for CR-LPWAN-based network architectures, briefly highlighted as follows:

#### 5.3.1. CR at the LPWAN End Node

In CR at the LPWAN end node (CREN) scheme, CR is embedded in the LPWAN technology deployed in different IoT end nodes; thus, such end nodes, for example, IoT sensors such as smart meters, are responsible for executing all CR functions. In this scheme, IoT end nodes are designed to be robust and complex to conduct SS, decision making, mobility, and spectrum access. This may impact on cost of the end node, and increase the power consumption rates of such end nodes; nevertheless, it reduces the network overhead since end devices and GWs would communicate less in terms of channel convergence and control messaging. As an example, the CREN scheme was adopted in [46] wherein end nodes were embedded with a channel manager for decision making, spectrum detection, and prediction.

#### 5.3.2. CR at Gateway

In CR at gateway (CRGW) scheme, CR technologies are embedded in the GW. In this case, the CR-LPWAN GW conducts all CR functions (particularly the spectrum and decision making functions) and communicates the acquired white spaces to all end nodes in the network. Then, end nodes only conduct spectrum access/sharing. This may present certain challenges for adapting the CRGW scheme, for example, in the Sigfox and LoRaWAN class A brands since both technologies imply ALOHA-like access methods. Thus, the GW can only communicate with an end node after the end node has sent some data in the uplink and only in a one-to-one mode. Additionally, Sigfox has very strict limits for the number of downlink packets sent. Thus, it may be challenging to implement the CRGW scheme, since this may require modifications to the existing respective protocols, for example, the need to depart from LoRaWAN Class B/C technologies. Nevertheless, it is envisaged that the CRGW scheme will reduce the design complexities of the end nodes, which will reduce the device costs of CR-LPWAN systems and the power consumption rates of end nodes. However, network message overheads may increase owing to the need for continuous negotiations between the GW and the large number of end nodes in the network. These issues are potential research issues to be addressed towards developing functional CR-LPWAN systems. An example of the CRGW scheme can be found in [10].

#### 5.3.3. CR at both LPWAN End Node and Gateway

In CR at both end node and gateway (CRENGW) scheme, all devices in the network are embedded with CR technologies. In this case, all devices, including end nodes and GWs, perform all CR functions including sensing white spaces, making decisions, and accessing the network. An additional function that could be performed in CRENGW is cooperative sensing, wherein IoT end nodes interact to decide pertaining to the presence/absence of PU signals. CRENGW is more complex in its design as against other schemes, however, it provides the highest communication reliability use-case, which makes it suitable for sensitive applications in medical and military domains.

Following the above schemes, and similar to [76], we prescribe the CRGW scheme in the architecture of Figure 5. Deploying CR functions at the GW instead of at the end-nodes may be appropriate since it minimizes design complexities at the LPWAN end nodes, which are often resource constrained. It is convenient to deploy CR technologies at the GW since most GW infrastructures are designed to be robust both computationally and memory wise. Furthermore, GWs are often connected to power supply utility outlets and to backup power supply units, thereby assuaging the increased energy demands of integrating CR in LPWAN.

When in operation, the CR-LPWAN GW scans the electromagnetic spectra to detect white spaces. In other designs, the geolocation database technology was used at the GW [85]. However, apart from being motivated by the IEEE 802.22 standard for CR [86], we suggest that SS be deployed in CR-LPWAN systems, because a number of IoT applications/devices are designed to be deployed in remote locations wherein internet services may be unavailable. In such a case, it becomes ineffective to rely on geolocation technologies.

When a free channel is detected, the CR-LPWAN GW sends such free channels to all LPWAN end nodes in the network, which will be used by end nodes for onward communication to the GW and subsequent IoT devices. The GW may be linked to an IoT network server (NS) connected to different application servers (AS) based on the application area through a firewall. To improve access, most GWs may connect either via wireless or wired links to the internet. Although simple, the architecture of Figure 5 facilitates the concept of CR-LPWANs for IoT applications. Future ideas can be developed in this regard.

## 6. Benefits of CR-LPWAN

CR-LPWAN systems will be beneficial to many IoT applications and we discuss a number of these benefits in two categories. First, we discuss general technical benefits of CR to LPWAN, and then we discuss a few application areas that may benefit from CR-LPWAN systems. These are highlighted as follows:

### 6.1. Technical Benefits to IoT Devices

CR-LPWAN presents a number of technical benefits to IoT at both the device and network level, which we discuss as follows:

#### 6.1.1. Improved Spectral Utilization

Basically, CR-based technologies are designed to detect the presence/absence of PU signals in a licensed band. The absence of PU signals in a licensed band provides for opportunistic communication, whereas the presence of PU signals signifies the need for immediate rescission from the band. Such a novel communication paradigm aims to improve the use of underutilized bands leading to improved QoS experiences for end users. An example of a CR-LPWAN that provides for improved spectral utilization is the sensor network over white spaces (SNOW) solution, which takes advantage of white spaces in the TV band for opportunistic transmission [87]. Thus, the use of CR is promising as it will guarantee less constraints on the channel bandwidth size of LPWAN technologies, which will ultimately improve their capacity for higher data rates [85].

#### 6.1.2. Less Transmission Power Constraints

LPWAN technologies are able to achieve longer transmission range by increasing their transmission power at the expense of smaller signal bandwidths [4]. Nevertheless, transmit power levels are constrained because of stringent regulations in the ISM band pertaining to duty cycle and interference-imposed constraints. Thus, incorporating CR in LPWAN will allow the use of white spaces that help to evade undue transmission power constraints, and ensure that licensed spectrum owners are not interfered with.

#### 6.1.3. Longer Transmission Range

Dynamic spectrum access implies that CR-LPWAN technologies will be able to leverage the opportunity to increase their transmission power leading to longer transmission ranges. This is possible in the VHF bands wherein signals are known to propagate much farther in space. This advantage is presently explored by Sigfox, which enables their networks to cover extremely long transmission ranges as against other LPWAN technologies. Said longer transmission range of Sigfox has been verified in practical deployment environments and test campaigns [88].

#### 6.1.4. Increased Scalability

The ability to seamlessly switch to licensed spectra will create additional bandwidth for LPWAN use. This increased bandwidth ultimately enables more end devices to transmit. At the moment, using a typical 200 kHz bandwidth and message bandwidth of 100Hz, Sigfox networks can accommodate over 2000 IoT devices at a single transmission time. In fact, by deploying CR, it is noted that Sigfox networks may service up to 1 million end devices per base station [43], which is similar to Nwave, which can accommodate up to 1 million devices per base station [43]. Furthermore, the Ingenu technology used in Dallas/Forth Worth, US, serves more than 4.4 million people using only 17 access points [43]. This large number of users per base station can be further up-scaled by developing and adopting CR-LPWAN systems.

#### 6.1.5. Improved Reliability

With less hardware involvement and additional SDN usage for CR purposes, CR-LPWAN systems will provide reliable network operability [85]. This implies that channel conditions will be better with free bands to use and additional spectrum characterized by lower noise levels as against the ISM bands. With potential diversity in the available spectral pool for CR-LPWAN technologies, the chances of using reliable channels will increase, thereby improving the QoS of LPWAN users.

### 6.2. Benefits to IoT Application Areas

#### 6.2.1. Smart Grids

Future energy production and supply networks will be based on the concept of smart grid networks (SGN). In addition to being more sophisticated than traditional energy supply methods, SGNs adopt complex communication networks in order to control and manage energy supply. In this use-case, SGNs use smart metering concepts to manage consumer demand and supply. Consequently, there exists constant communication between energy meters at consumer sites and at control facilities managed by the energy utility company. The network architecture for SGNs consists of three layers namely

1. Communication between smart meters and devices in the smart home called the home area network (HAN);
2. Communication between HAN and a network gateway called the field area network (FAN);
3. Communication between different HANs called the neighborhood area network (NAN)

CR-LPWAN systems can be deployed in FANs and probably in NANs. Since network gateways are located at great distances from most households, LPWANs can be used for long-range communication. CR in LPWAN can be beneficial under many conditions, for example, it could help to avoid interference between HANs, FANs, and NANs by adopting different frequency bands for each network. In this case, the NAN can be allocated white spaces in the VHF to enable long distance transmission since signals in the low VHF bands propagate much farther in space. CR-LPWANs could also provide increased bandwidths to ensure that utility companies query a large number of energy meters within larger communities at greater transmission speeds.

In the literature, we found some classic use of CR in smart grids, for example, in [46] a CR system architecture, algorithms and a hardware testbed were developed for SGN. They optimized the computing power and response latency of CR-SGNs. It was demonstrated that CR-SGN was able to recover data from simultaneous smart meter transmissions in the presence of strong wideband interference. Similarly, in [89], an energy harvesting approach was proposed for resource-constrained IoT devices. The node architecture of the energy harvesting CR comprised a power provisioning unit (PPU), an ultra-low power processing unit (ULPP), and an ultra-low power communication unit (ULPC). In the PPU, energy is harvested, rectified, regulated, and used to supply the ULPP and ULPC. The ULPP conducts CR SS, modulation, coding, and smart grid related security processes. The ULPC comprises the communication front-end including a demodulator, decoder, and other CR functions. The architecture in [89] adopts the CREN scheme (recall Section 5.3).

#### 6.2.2. Smart Homes

Many IoT applications are designed to automate home devices, wherein smart devices such as laptops, phones, TVs, cameras, domestic appliances, and smart meters are capable of self-organising and relaying information to users [28]. These IoT applications are presently deployed in the ISM bands, which implies that they must compete with existing ISM band devices. Competition causes further congestion of the crowed ISM bands leading to increased collision rates and poor link quality. Thus, there is need for IoT applications to switch from ISM bands to TV white spaces (TVWS) in order to improve network performance and reduce energy consumption rates in smart home applications [90]. Although intercommunication between home appliances are disposed to short range communication technologies like ZigBee, nevertheless, there are examples where CR-LPWAN may suffice. For example, smart cookers, alarm doorbells, and event switches can be remotely control by users at a distance. In this case, CR-LPWANs may be beneficial by enabling each device to migrate easily to white spaces that guarantee the best QoS.

In the literature, the application of CR in smart homes was discussed in [21,28,74]. For example, Rawat et al., in [18,28], discussed the use of CR to facilitate opportunistic communication in smart home devices via TVWS. In [91], CR access to TVWS was proposed supporting high definition TV (HDTV) distribution in smart homes. Sudha et al. [92] also discussed the use of CR in smart home environments and a prototype was developed, wherein National Instrument Universal Software Radio Peripheral (NI USRP) hardware platforms and LabView software were used to conduct experiments. These application use-cases further strengthen the potentials of deploying CR-LPWAN systems for smart home applications.

#### 6.2.3. Telemedicine

Smart sensors are deployed for telemedicine purposes to provide remote healthcare and medical services to outpatients. Telemedicine is driven by the need to provide realtime access to vital signs such as temperature, blood pressure, oxygen levels, and electrocardiogram signals of patients. This may involve implanting sensors in patients so that signals obtained via these sensors are communicated to a remote server situated at the doctor’s station. CR-LPWAN systems can be deployed to improve access to spectrum in telemedicine applications. Since vitals are critical datasets, it is essential to avoid errors and to guarantee high speed transmission. CR-LPWAN is well suited in such a case to provide low latency transmission in appropriate white spaces. Furthermore, in case of poor weather conditions causing increased error rates, CR allows LPWAN technologies to adapt modulation schemes to the appropriate band for improved performance.

In [93], an infrastructure-based CRN was introduced for telemedicine, wherein CR base stations were deployed to sense for free bands and forward such sensed data to a remote healthcare station. Support for real-time periodic telemonitoring of network traffic was also introduced. A CR system was developed in [94] for e-health applications in a hospital environment. The proposed system aimed at protecting medical devices from harmful interference by adjusting device transmission power to reduce electromagnetic interference. CR has been widely applied in wireless body area networks (WBAN) to improve medical services. For example, in [95], a viable CR architecture for medical body area networks was proposed based on ultra wideband radio technology. Further discussion on the use of CR in medical IoT applications can be found in [28,96,97,98]. Such classic examples indicate that CR in LPWAN is a promising venture for IoT applications.

#### 6.2.4. Vehicular Networks

The application of CR-LPWAN in vehicular networks presents great potentials for improved performance. CR-LPWANs would allow moving vehicles to communicate over long single hops to a remote roadside infrastructure against short range technologies, which require multi-hops to reach an infrastructure [99]. Specifically, high density vehicular networks could arise in market places, cinemas and sporting arenas where large events are ongoing. This high-density condition typically breeds increased interference, which might grow spontaneously to cripple the entire network. CR-LPWAN systems are most suited to such cases by providing frequency hopping capacity to robust bands in order to relieve network congestion and improve performance.

In the literature, for example, CR was used to increase communication bandwidth in vehicular ad hoc networks [100]. In particular, cooperative SS was deployed among neighboring vehicles along with a belief propagation model. This was applied to address the problem of distributed observations and to exploit redundancies in both space and time. Improved performance was recorded. A spectrum database assisted CR vehicular network design was proposed in [101], which adheres to the FCC specification for CR deployment, and it also provides a cost-revenue analysis based on parameters such as vehicular density, base station road coverage, and database assisted sensing. The findings in [101] will greatly improve the development of CR in vehicular networks in terms of cost reduction and long-range transmissions. There are several applications of CR in vehicular networks, which can be found in [28,102], and CR-LPWAN can only add to this growing list of application benefits.

#### 6.2.5. Smart Agriculture

Smart agriculture could involve deploying sensors on foraging animals in order to monitor and keep track of their movement and health conditions [103]. Since these animals often transverse long distances, CR-LPWAN systems could provide white space transmission to achieve longer range communication. Most importantly, CR-LPWAN systems would allow these sensors to transmitat different frequency bands to avoid interference particularly when they forage faraway to uncharted terrains.

## 7. Challenges/Future Directions of CR-LPWAN

We categorize challenges in CR-LPWAN into challenges at the PHY and upper layer levels. Often, challenges at the PHY layer are known CR problems, which determine to a large extent the success of CR-LPWAN systems, whereas challenges at the upper layers are typical problems capable of constraining the efficacy of CR-LPWAN systems. These are discussed as follows:

### 7.1. PHY Layer Challenges

A number of PHY layer related challenges are discussed as follows:

#### 7.1.1. Rendezvous

The rendezvous problem is related to the realization of a common control channel (CCC) for control messaging in CR-LPWAN networks and may be experienced in ad-hoc-based CR-LPWAN network configurations where coordination will be required between end nodes. Rendezvous defines the time it takes for two or more CR end nodes to converge on a CCC. In practice, it is challenging for CR-LPWAN nodes to simultaneously agree on a free channel to be used as CCC for coordinating communication between CR-LPWAN end nodes. This challenge occurs because the probability of two CR-LPWAN nodes scanning and converging on a CCC without prior knowledge or agreement between both entities is often very low. This is because different CR-LPWAN nodes often start their search for white spaces at different frequency points, and may find these white spaces at different bands of the RF spectrum. Thus, it is often difficult for two CR-LPWAN nodes to start and discover the same white space at that same time and frequency location. This challenge is sometimes called the “CCC setup challenge” [28].

There are a number of efforts geared towards addressing this challenge such as in [104,105,106,107,108,109,110,111,112,113]. For example, in [104], a rendezvous system was proposed wherein end nodes search for free bands without any coordination. The developed system contributed mainly in extending rendezvous convergence to multiple users by inheriting hopping sequences from other users. Their distributed algorithms called coordinated channel hopping (CCH) achieved 80% lower time to rendezvous than existing algorithms. Similarly, while noting that implementing rendezvous is challenging since CR end nodes are unaware of each other before rendezvous, authors in [112] proposed a fast rendezvous algorithm based on channel hopping. It was noted that the proposed fast rendezvous algorithm can guarantee rendezvous in $2N^{2}+N$ timeslots under asynchronous environments, where *N* is the number of licensed channels in the network. Ohize and Dlodlo in [113] proposed a channel hopping algorithm for rendezvous in CRN using swarm intelligence. They deployed an ant colony optimization algorithm to guide nodes towards converging on the best channel.

Essentially, we note that the literature on the rendezvous problem in CR is quite rich, and it is a contemporary challenge that would affect CR-LPWAN systems. As such, it is pertinent to address the rendezvous problem towards developing effective CR-LPWAN systems. Specifically, further research is required to ensure that rendezvous algorithms are fast, simple, and energy efficient for CR-LPWAN purposes.

#### 7.1.2. Spectrum Sensing

Spectrum sensing (SS) is a fundamental function of any CR system including CR-LPWAN systems. SS determines the presence/absence of PU signals in any band of interest. This is achieved by comparing the estimated energy value in a channel with a threshold value. If the energy value surpasses the threshold, then a PU is declared present, and if the energy value is below the threshold, the band is declared free (white space). There are several methods proposed for SS in CR including: energy detection (ED) method [20,29,114,115,116,117,118], matched filter, cyclostationary detection, eigenvalue method, covariance method, and prediction-based approaches [72]. Other recent applications of evolutionary algorithms to improve SS performance in CR have been identified [119,120,121,122].

There are several challenges in developing effective and efficient SS techniques for CR-LPWANs. For example, the ED is a fast, simple and cost effective method for SS, however, it performs poorly in low SNR regimes [117,123,124]. This poor performance occurs because the ED depends on predefined threshold values for detection. Many CR-LPWAN systems may be deployed in critical application domains such as military, medical, and business domains. These application areas require greater accuracy levels, which the ED may be unable to render. For example, in low SNR conditions, the threshold value of the ED drops too close to the noise floor resulting in increased noise samples crossing the threshold value, leading to increased false alarm rates. There are several options to address this challenge, for example, using effective adaptive threshold techniques such as the forward consecutive mean excision (FCME) algorithm [125,126], double threshold methods [110,117,118,127,128,129,130], Otsu [114,124], recursive methods [124,131], and several other methods [132,133,134,135,136,137]. These methods would compute new threshold values without prior knowledge of the noise floor in order to prevent excessive false alarms, and to maximize the detection rate of CR-LPWANs. Furthermore, it is essentially to reduce the computational requirements and the energy consumption rates of ED methods deployed in CR-LPWAN systems.

#### 7.1.3. Local or Cooperative Sensing

Local sensing is conducted by a single node in order to decide whether PU signals are present or absent in any band of interest. On the other hand, cooperative sensing involves multiple end nodes conducting SS and collectively deciding whether PU signals are present or absent [138]. This decision is often taken by a central coordinator. There are different approaches for cooperative sensing including linear combination of local sensing from individual nodes [139], k−out−of−N approach wherein a certain number of participating nodes is required to conclude on a certain outcome [140], the And-method in which any node that declares a band free (null hypothesis) would make the coordinator to declare the band as free [141]. Cooperative sensing is theoretically more effective than local sensing as it alleviates the issues of shadowing and fading due to the collaborative efforts from different nodes [138]. However, cooperative sensing introduces complexities, such as large messaging overhead between nodes and gateways, and poor energy efficiency across nodes. Local sensing on the other hand is limited by errors due to shadowing and fading. Thus, it is essential to develop new methods to address these limitations for effective CR-LPWAN deployment.

#### 7.1.4. Spectrum Mobility

Spectrum mobility enables CR systems to migrate seamlessly from an occupied channel to a free band without disrupting ongoing communication sessions. It is often called spectrum hand-off [54]. One main challenge in spectrum mobility is the presence of temporary breaks during communication, which will greatly hamper the effectiveness of CR-LPWAN systems as well. Although glitches in communication are inevitable, nevertheless, it is essential to develop new methods for spectrum mobility that would reduce such degrees of discontinuities to the barest minimal.

#### 7.1.5. Incorporating Adaptive CR Technologies

LPWAN transceivers can be integrated with adaptive CR technologies. For example, the SX1276 LPWAN transceiver is a product of Semtech’s patented LoRa technology [142], which features the LoRa long-range modem and provides high immunity to interference, on top of ensuring minimal current consumption rates. An area where adaptive CR technology can be integrated is in the on-off keying (OOK) modulation module of the SX1276 transceiver. The OOK demodulator in the SX1276 chip works by comparing the RSS output with a threshold value [142]. It uses three different threshold modes, including the “manual or fixed,” “peak,” and “average” threshold methods, all configured using the OokThreshType instruction set [142].

Towards incorporating CR technologies, it is required that these threshold methods be optimized to ensure that licensed signals are accurately detected. The use of adaptive threshold estimation algorithms (ATAs) is advocated in the OOK demodulator block of the SX1276. There are several ATAs available for use such as the FCME algorithm [143], the recursive onesided hypothesis testing (ROHT) algorithm [124], the first order statistical technique (FOST) [144] also called the m-dB method [145], and several other ATAs [128,132,146]. Nevertheless, one research issue to be addressed is the reduction in the computational complexities of these ATAs in order to reduce the power consumption rate of the SX1276 chipset. Summarily, adaptive CR technologies such as ATAs are essential in LPWAN transceivers in order to improve spectrum identification, reduce interference, and ensure effective bandwidth utilization. Some transceiver chipsets such as the ADF7241 based on IEEE 802.15.4 Zero-IF standard [147], the ATA8520 chip by Sigfox [148], the ASIC transceiver by Weightless, and the NANO-S100 by Ingenu [149], use the “manual or fixed” threshold method, and as such, they will largely benefit from adaptive CR technologies.

#### 7.1.6. Other Challenges

Other PHY layer challenges that may confront the development of effective CR-LPWAN systems are listed as follows:

1. Improving scalability to admit additional devices;
2. Control and mitigation of interference issues between CR and PU operators [4,5];
3. Development of improved and higher data rate techniques for CR-LPWAN systems [4,5];
4. Interoperability between different CR-LPWAN technologies;
5. Localization, link optimization, and adaptability issues [150];
6. Developing functional testbeds and simulators;
7. Security, mobility, roaming, and co-existence issues with other wireless technologies [4,5];
8. Support for data analytics, efficient spectral utilization, and reliability [5].

### 7.2. Upper Layer Challenges

It is noted that CR is a major PHY layer solution to different communication networks including the LPWAN. Nevertheless, there are unique challenges that may limit the efficacy of CR-LPWAN systems, which we discuss as follows:

#### 7.2.1. Connectivity Challenges

Here, we highlight challenges that may prevent CR-LPWAN devices from establishing connection in CR-LPWAN networks as follows:

1. **Connectivity compatibility issue:** The challenge of connectivity compatibility, also termed interoperability issue, stems from conditions wherein different users adopt different connectivity technologies for different CR-LPWAN systems. This is due to the present large IoT market space, wherein technologies are developed from different vendors. These technologies are often proprietary in nature, which compounds further the issue of compatibility. It is thus difficult for users to migrate easily or interconnect devices obtained from different vendors. This will cause a divide in the market, which will ultimately limit the range of CR-LPWAN systems and stifle user satisfaction.
2. **Maintainability issue**: With different products being available for use, different CR-LPWAN systems will have different reliability and durability levels. Consequently, devices may develop faults, shut down, or batteries may expire at different rates, limiting connectivity in CR-LPWAN systems. In such cases, maintenance should be made easy to reduce the length of down times.
3. **Signalling**: Different technologies have different communication modes, which may hamper signalling between CR-LPWAN end nodes and different gateways. For example, bidirectional signalling may be required to ensure effective data delivery in CR-LPWAN networks. However, due to signalling mismatch between different CR technologies, there may be breakdown in connectivity.
4. **Bandwidth**: Bandwidth usage and management is a factor in ensuring effective connectivity in CR-LPWAN networks. With different products available for IoT applications, there may be more bandwidth requirement in an application over another. Thus, inability to meet minimum bandwidth requirements across a variety of applications may cause increase transmission error rates, which will limit connectivity.
5. **Transmission Power levels**: Increasing transmission distance often depends on the transmission power level of CR-LPWAN end nodes. Thus, deploying devices with disparate transmission power levels can hamper the connectivity of CR-LPWAN networks. Consequently, effort is required to develop CR-LPWAN nodes that meet specific power transmission levels in order to ensure effective information transfer over CR-LPWAN networks.

#### 7.2.2. Networking Challenges

Similar to most networks, CR-LPWAN networks are plagued by challenges at the networking level. Owing to the large internetwork within the IoT space, quite a number of networking protocols exist to ensure seamless interaction and transfer of data. Most IoT applications interact with sensors and actuators in order to monitor and conduct different tasks. Consequently, careful CR-LPWAN network designs are required to ensure efficiency and scalability of such networks. In this regard, a few challenges at the networking level are discussed as follows:

1. **Small packet sizes:** The requirement for low power consumption rates along with long transmission ranges may limit the data frame size to be deployed in CR-LPWAN networks. For example, the data frame size for Sigfox technologies is a total of 26 bytes [151], whereas a total payload of 222 bytes (for data rates 4–7) is provided by LoRa for use in Europe and 242 bytes for use in the US [152]. This is quite small to satisfy the demands of all IoT use-cases. Furthermore, there are different standards for frame sizes across different proprietary technologies, which may render the networking process of CR-LPWAN devices a burdensome task. Improvement in the standardization process is highly encouraged.
2. **Routing**: Many CR-LPWAN networks consist of large numbers of end nodes; thus, effective routing schemes are essential. In this regard, light weight routing protocols are required in CR-LPWANs since CR functionalities often incur additional computational overheads. The IETF is strongly involved in addressing routing issues in IoT with several new protocols being deployed, for example, the CoAP. They have also developed the RPL (IPv6 Routing Protocol for Low-Power and Lossy Networks), which tackles problems associated with mesh networking in IoT [153]. With the burgeoning of CR-LPWAN, these standards may require further extensions in order to address the challenge of increased routing computational demands, which may arise from spectrum mobility across different white spaces.

#### 7.2.3. Security Challenges

IoT applications often involve the transfer of confidential information, for example, acquiring medical vitals in telemedicine, or within smart city networks, wherein utility bills are transferred. These applications may require secured links before communication can be initiated. This may be realized, albeit at the expense of increased complexities, energy consumption rates, and costs, which will limit many IoT use-cases [154]. Thus, attacks such as unauthorized use of personal data, data leaks or spoofing, hacking, denial of service, and flooding, are potential challenges that must be addressed in CR-LPWAN networks. In fact, the use of CR in LPWAN introduces the specific security problem of primary user emulation (PUE) attack, wherein attackers could emulate bona fide PU transmitters in order to deprive other CR-LPWAN devices access to white spaces [155]. Other specific security challenges may include:

1. **Light weight Security Protocols:** There are a number of existing security protocols for traditional wireless networks and IoT networks [156], which cannot be deployed for CR-LPWAN applications because of their complexities. It is thus required to develop light weight security protocols to ensure low power consumption rates, and reduced complexity in CR-LPWANs.
2. **Software Vulnerability:** It is often expected that the initial version of most software are plagued by programming bugs known as software vulnerabilities [154]. It is essential to reduce as much as possible the number of bugs in CR-LPWAN software, e.g., spectrum mobility and management software, to reduce the risk of back-door sabotage, which may lead to malicious attacks and network downtimes if left unchecked.
3. **Malware:** The promising and open spectrum-access nature of CR-LPWAN suggests that it is a potential breeding ground for malware attacks. Malicious attackers are constantly on the prowl looking for possible loopholes to exploit. While malware attacks against IoT software may seem far fetched, it is worth noting that Symantec has declared the detection of the first piece of IoT malware (a worm) termed “Linux.Darlloz” [157]. Others include Mirai, which turn networked devices running Linux into remotely controlled bots for large-scale network attacks. These discoveries imply that CR-LPWAN devices and networks are not exempted from possible malware attacks, which demand new research measures to improve security.

## 8. Conclusions

Low power wide area networks (LPWANs) are being widely deployed to interconnect many IoT-based applications, particularly applications that require long transmission ranges, low device/development costs, low power consumption rates (i.e., long battery lifetimes), and high scalability, albeit at low data rates. Nevertheless, many of these LPWAN technologies are frequently susceptible to a number of persisting challenges, such as increased interference in the ISM band, spectral inefficiency, and low data rates. Consequently, there is need for new ideas and paradigms to address these problems for which cognitive radio (CR) suffices as a candidate solution. Thus, in this article, we have discussed the concept of deploying CR in LPWAN systems via a review of different state-of-the-art developers in LPWAN technologies, their respective competing standards, and several application areas; likewise for CR systems. Details regarding the development and integration of CR in LPWAN for IoT were considered on a general note, including the description of a simple network architecture for CR-LPWAN, potential benefits, and challenges. Essentially, this paper has examined the possibilities for CR-LPWAN systems. Presently, only a few LPWAN technologies provide full support for CR in their technologies; thus, this article intends to spur future research interests in the study area of CR-LPWAN systems.

## Figures and Tables

**Table 1:** Timeline of the origins of different LPWAN technologies.

S/N	LPWAN Technology	Developer/Company	Year of Origin
1	MOBITEX	Televerket Radio	Beginning of 1980s
2	ARDIS/DataTAC	Motorola	1980–1990s
3	AlarmNET (ADEMCO)	Motorola	1990–2000s
4	GPRS	Cellular Network Developers	2000s
5	IQRF	IQRF	2004
6	Telensa	Telensa (Formerly part of Plextek Inc.)	2005
7	RPMA	Ingenu (Formerly known as OnRamp)	2008
8	Sigfox	Sigfox	2009
9	Qowisio	Qowisio	2009
10	Wi-SUN	Wi-SUN	2011
11	LoRa/LoRaWAN	LoRa Alliance	2012
12	Weightless	Weightless SIG	2012
13	Symphony Link	Link Labs	2013
14	Adaptrum	Adaptrum	2014
15	NB-IoT/LTE-M	3GPP group	2016

**Table 2:** Ranking of the different LPWAN brands based on their potential integration of cognitive radio (CR) technologies.

Rank	LPWAN Technology	Licensed Band Operation	Unlicensed Band Operation	Currently Supports CR	Supports Multiple Channels	Considering Future CR Deployment
1	Nwave	✓	✓	✓	✓	✓
2	Sigfox	✕	✓	✕	✓	✓
3	Weightless	✕	✓	✓	✓	✓
4	LoRa	✕	✓	✕	✓	✓
5	Symphony Link	✕	✓	✕	✓	✓
6	Amber Wireless	✕	✓	✕	✓	✕
7	IQRF	✕	✓	✕	✓	✕
8	LTE-M	✓	✕	✕	✓	✕
9	NB-IoT	✓	✕	✕	✓	✕
10	Starfish	✕	✓	✕	✓	✕
11	Telensa	✕	✓	✕	✓	✕
12	Wi-SUN	✕	✓	✕	✓	✕
13	Qowisio	✕	✓	✕	✕	✕
14	Ingenu	✕	✓	✕	✕	✕

**Table 3:** Summary of the different standard development organizations.

S/N	SDOs	Overview	Area of Focus	Compliant LPWAN Technologies	Number of Participating Members & Organizations
1	IEEE 802.15.4	Addresses protocol development and compatible interconnection for devices requiring low data, low power, low complexity and short range transmission	(1) PHY Layer consideration: QPSK, BPSK, ASK, CSS, UWB, GFSK (2) MAC Protocol development (3) Security: Lookup procedures, security operations and header	Zigbee, Bluetooth, Wi-SUN, Sigfox, Symphony, Ingenu RPMA	∼216 (Corporate Members)
2	ETSI	Developing LTN for long-range data transportation, long battery lifetime, high scalability and low throughput services	(1) Application areas such as smart metering, smart cities, automotives, e.t.c (2) Network topology (3) Traffic and Protocol harmonization (4) Identifiers and addressing (5) Security aspects (6) End point implementation	Sigfox, LoRa, Silver Spring, Telensa	>400 (Individual Members)
3	3GPP	Provision for low power consumption, low device cost, improved outdoor and indoor penetration, optimized data transfer, scalability for capacity upgrade	(1) Architecture enhancement for MTC (2) Addressing (3) Identifiers (4) Device triggering (5) Small data enhancement (6) Power consumption rate (7) Battery saving (8) Monitoring enhancement	NB-IoT, LTE-M, EC-GSM-IoT	>800 (Including Individual and Corporate Members)
4	IETF	Interested in enabling a wide range of things to use interoperable technologies including for the IoT including covering technologies surrounding LPWAN characteristics	(1) Header compression (2) Fragmentation (3) Reassembly (4) Management (5) Security, Integrity, and Privacy (6) Neighborhood discovery	LoRaWAN, NB-IoT, Sigfox, Wi-SUN FAN	Involuntary membership

**Table 4:** Summary of the different special interest groups.

S/N	SIGs	Focus	Open	Non-Profit based	Number of Members	Support for Cognitive Radio
1	LoRa Alliance	To standardize LPWAN for IoT applications, and also to drive the global success of LoRa protocol for interoperability between operators	✓	✓	>130 (mainly companies)	Not Yet
2	Weightless SIG	To coordinate and enable all activities required to ensured interoperable standards for wide area IoT connectivity	✓	✓	∼4752 Individual members	Yes
3	Dash Alliance	Development and enhancement of DASH7 protocol specification and other DASH7 technologies for global adoption by national and international standard bodies/agencies	✓	✓	9 (These are mainly companies excluding the number of students/Professors from 4 Universities)	Not Yet
4	IQRF Alliance	Deliver interoperable wireless IoT devices and solutions for fast realization of wide range of IoT projects	✓	✓	∼98 (Including 45 Institutions, 45 Adopters, and 5 Contributor companies)	Not Yet
5	Wi-SUN Alliance	To drive the global proliferation of interoperable wireless solutions for IoT applications using global open standards	✓	✓	176 (Including 87 contributor companies, 79 Adopter companies, and 10 Promoter companies)	Not Yet
6	IoT World Alliance	To deploy IoT solutions seamlessly worldwide through a single point of contact. Ensure the use of a Single SIM world wide while reducing the cost of data connectivity	✕	✕	∼70 (mainly companies)	Not Yet

**Table 5:** Overview of the different standardization-based consortia working towards IoT standardization.

S/N	Consortium	Focus	Number of Members	Open Membership	Non-Profit	Annual Dues Required
1	Oasis IoT	Technology Architecture Focused (TAF): Building protocols such as AMQP, MQTT, oBIX	∼5000)	✓	✓	✓
2	Object Management Group	TAF: Developing Data distribution services and also managing the IIC	∼327	✓	✓	✓
3	Open Interconnect	Providing software support including platform support of different Operating Systems. They are also defining connectivity requirements for interoperability of IoT devices	∼150	✓	✓	✓
4	Industrial Internet	Works with the Object Management Group to catalyse, coordinate and enable growth of the Industrial Internet. They work on Data Distribution Services, and unifying component models for real-time and embedded systems	∼293 (mainly companies)	✓	✓	✓
5	Internet of Things	Ensuring the global adoption of IoT products and services through research and market education	∼50	✓	✓	✕

## Abbreviations

The following abbreviations are used in this manuscript:

3GPP	3rd Generation Partnership Project
6LoWPAN	IPv6 for low power wireless personal area networks
ADEMCO	alarm device manufacturing company
AES	Advance Encryption Standard
ARDIS	advanced radio data information service
AS	application server
ATA	adaptive threshold estimation algorithm
BPSK	binary phase shift keying
CCC	common control channel
CR	cognitive radio
CREN	cognitive radio at end node
CRGW	cognitive radio at gateway
CRENGW	cognitive radio at both end node and gateway
CRN	cognitive radio network
CRS	cognitive radio system
CRSN	cognitive radio sensor network
CSMA/CA	carrier sense multiple access with collision avoidance
CSS	chirp spread spectrum
CD	cyclostationary detector
D-BPSK	differential binary phase shift keying
DC	direct current
DECT	Digital Enhanced Cordless Telecommunication
DSSS	direct sequence spread spectrum
E2E	End 2 End
EC-GSM-IoT	extended coverage GSM IoT
ED	energy detector
eDRx	enhanced discontinuous reception
eMTC	enhanced machine-type communication
ETSI	European Telecommunications Standards Institute
FAN	field area network
FCC	Federal Communications Commission
FCME	forward consecutive mean excision
FDMA	frequency division multiple access
FEC	forward error correction
FFT	fast Fourier transformation
FDMA	frequency division multiple access
FOST	first order statistical technique
FSK	frequency shift keying
GMSK	Gaussian minimum shift keying
GSM	Global System for Mobile communication
GW	gateway
HAN	home area network
HDTV	high definition television
HTTP	Hypertext Transfer Protocol
IEEE	Institute of Electrical and Electronics Engineers
IETF	Internet Engineering Task Force
IF	intermediate frequency
IFA	inverted F antenna
IIC	Industrial Internet Consortium
ISM	industrial, scientific, and medical
IoT	Internet of Things
IP	Internet Protocol
IPSO	Internet Protocol for Smart Objects
ITU	International Telecommunication Union
LECIM	low energy critical infrastructure monitoring
LNA	low noise amplifier
LoRa	Long Range
LPWAN	low power wide area network
LRLP	long-range low power
LTE-M	Long-Term Evolution, category M1
LTN	low throughput network
MAC	medium access control
NAN	neighbourhood area network
NB-IoT	Narrowband IoT
NS	network server
OFDMA	orthogonal frequency division multiple access
OOK	on-off keying
OSSS	orthogonal sequence spread spectrum
PCA	priority channel access
PHY	physical
PPU	power provisioning unit
PSM	power saving mode
PU	primary user
PUE	primary user emulation
QAM	quadrature amplitude modulation
QoS	quality of service
QPSK	quadrature phase shift keying
RF	radio frequency
ROHT	recursive onesided hypothesis testing
RPMA	random phase multiple access
RSS	received signal strength
SC-FDMA	single carrier frequency division multiple access
SDN	software-defined network
SDO	standard development organizations
SDR	software-defined radio
SDWSN	software-defined wireless sensor network
SG	smart grid
SGN	smart grid network
SGCN	smart grid communication network
SIGs	Special Interest Groups
SNOW	sensor network over white spaces
SNR	signal to noise ratio
SoC	system on a chip
SRD	short range device
SS	spectrum sensing
SSL	secured sockets layer
SUN	smart utility network
TG	task group
TVWS	television white space
UHF	ultra high frequency
ULPC	ultra-low power communication
ULPP	ultra-low power processing
UNB	ultra narrow band
USRP	Universal Software Radio Peripheral
VHF	very high frequency
WG	working group
Wi-SUN	wireless smart utility network
WBAN	wireless body area network
WLAN	wireless local area network
WSN	wireless sensor network
